# SARS-CoV-2 Seroprevalence in a Berlin Kindergarten Environment: A Cross-Sectional Study, September 2021

**DOI:** 10.3390/children11040405

**Published:** 2024-03-28

**Authors:** Julian Bernhard, Stefanie Theuring, Welmoed van Loon, Marcus A. Mall, Joachim Seybold, Tobias Kurth, Raquel Rubio-Acero, Andreas Wieser, Frank P. Mockenhaupt

**Affiliations:** 1 Institute of International Health, Charité Center for Global Health, Charité—Universitätsmedizin Berlin, Corporate Member of Freie Universität Berlin and Humboldt-Universität zu Berlin, 13353 Berlin, Germany; stefanie.theuring@charite.de (S.T.); welmoed.van-loon@charite.de (W.v.L.); frank.mockenhaupt@charite.de (F.P.M.); 2 Department of Pediatric Respiratory Medicine, Immunology and Critical Care Medicine, Charité—Universitätsmedizin Berlin, Corporate Member of Freie Universität Berlin and Humboldt-Universität zu Berlin, 13353 Berlin, Germany; marcus.mall@charite.de; 3 Berlin Institute of Health (BIH) at Charité—Universitätsmedizin Berlin, 13353 Berlin, Germany; 4 German Centre for Lung Research (DZL), 35392 Gießen, Germany; 5 Medical Directorate, Charité—Universitätsmedizin Berlin, Corporate Member of Freie Universität Berlin and Humboldt-Universität zu Berlin, 10117 Berlin, Germany; joachim.seybold@charite.de; 6 Institute of Public Health, Charité—Universitätsmedizin Berlin, Corporate Member of Freie Universität Berlin and Humboldt-Universität zu Berlin, 10117 Berlin, Germany; tobias.kurth@charite.de; 7 Division of Infectious Diseases and Tropical Medicine, Ludwig-Maximilians-Universität, 80802 Munich, Germanywieser@mvp.lmu.de (A.W.); 8 Max von Pettenkofer Institute of Hygiene and Medical Microbiology, Ludwig-Maximilians-Universität, 80336 Munich, Germany; 9 German Centre for Infection Research (DZIF), 80802 Munich, Germany; 10 Fraunhofer Institute for Translational Medicine and Pharmacology (ITMP), 80779 Munich, Germany

**Keywords:** kindergarten, daycare, SARS-CoV-2, COVID-19, antibody, seroprevalence, pre-school children, Germany

## Abstract

SARS-CoV-2 serology may be helpful to retrospectively understand infection dynamics in specific settings including kindergartens. We assessed SARS-CoV-2 seroprevalence in individuals connected to kindergartens in Berlin, Germany in September 2021. Children, staff, and household members from 12 randomly selected kindergartens were interviewed on COVID-19 history and sociodemographic parameters. Blood samples were collected on filter paper. SARS-CoV-2 anti-S and anti-N antibodies were assessed using Roche Elecsys. We assessed seroprevalence and the proportion of so far unrecognized SARS-CoV-2 infections. We included 277 participants, comprising 48 (17.3%) kindergarten children, 37 (13.4%) staff, and 192 (69.3%) household members. SARS-CoV-2 antibodies were present in 65.0%, and 52.7% of all participants were vaccinated. Evidence of previous infection was observed in 16.7% of kindergarten children, 16.2% of staff, and 10.4% of household members. Undiagnosed infections were observed in 12.5%, 5.4%, and 3.6%, respectively. Preceding infections were associated with facemask neglect. In conclusion, two-thirds of our cohort were SARS-CoV-2 seroreactive in September 2021, largely as a result of vaccination in adults. Kindergarten children showed the highest proportion of non-vaccine-induced seropositivity and an increased proportion of previously unrecognized SARS-CoV-2 infection. Silent infections in pre-school children need to be considered when interpreting SARS-CoV-2 infections in the kindergarten context.

## 1. Introduction

The role of children in SARS-CoV-2 transmission and their potential as reservoirs has extensively been discussed [1,2,3,4,5,6,7,8]. COVID-19 infections in young children tend to be mild or asymptomatic [8,9], and are severely underdiagnosed [10,11,12]. This partially explains why infection dynamics in the kindergarten context remain incompletely understood [13]. This context encompasses preschool children, their families and educational staff, each with distinct patterns of interpersonal interaction, vulnerability and risk of infection. The kindergarten environment presents unique challenges due to the widespread absence of routine virus screening and the difficulties associated with implementing mask-wearing or maintaining physical distance among young children [7,14]. Seroprevalence within families is higher when adult family members are index cases compared to when children are the index cases [15]. On the other hand, increased odds of infection have been observed in preschool educators as compared to teachers working with older children illustrating that infectious risk might also depend on child age and/or behavior [16]. Widespread vaccinations have further complicated the disentanglement of transmission routes and risk factors. Nevertheless, information on infection risks as reflected by infection prevalence, on potential pathways and risk factors as well as on manifestation in young children remain crucial for the preparedness in the face of potential resurgences or emerging pathogens. We aimed to provide descriptive data in this regard. Therefore, in the present study, we assessed (i) SARS-CoV-2 seroprevalence and (ii) the proportion of so far unrecognized SARS-CoV-2 infections in Berlin kindergarten children, kindergarten staff, and connected household members in September 2021. We further assessed (iii) associated factors with unrecognized SARS-CoV-2 infections and preceding infections.

## 2. Materials and Methods

**Figure 1:**
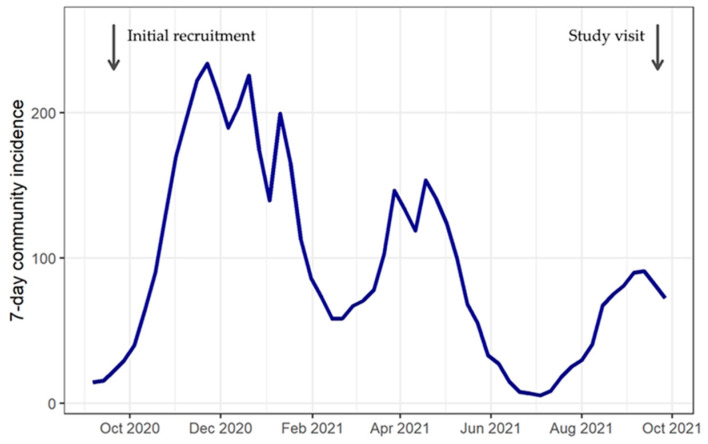
Community incidence in Berlin, 2020–2021.

This study analyzes cross-sectional seroprevalence data collected in Berlin in September 2021. Community incidence of SARS-CoV-2 infection in the preceding months varied greatly (Figure 1). The alpha variant (B.1.1.7) was replaced by the delta variant (B.1.617.2) starting in June 2021, reaching 99% by September 2021 [17]. Kindergartens were closed in the second lockdown in Germany between December 2020 and March 2021. This closure continued in Berlin until May 2021, except for children of essential workers such as health care professionals [18,19]. After reopening, kindergartens continued to follow distancing and hygiene rules. Wearing a facemask was mandatory only for contact between adults (staff and parents), but not for contact with and among children. In Germany, the first vaccinations against SARS-CoV-2 were available for adults from December 2020 following a prioritization scheme. From June 2021, children aged ≥12 years also had the opportunity to get vaccinated [20]. By September 2021, 68% of the German population had received at least one vaccination, whereas among children and adolescents aged 12–17 years, 59% were not yet vaccinated [21]. Vaccination against SARS-CoV-2 was not recommended for children of kindergarten age.

This study was conducted as part of the Berlin Corona School Study (BECOSS) with an initial recruitment phase in September 2020, as previously described [3]. Twelve kindergartens were randomly selected out of the more than 2700 facilities in Berlin. This selection process involved dividing the city districts into three strata according to socioeconomic status. Within each stratum, two districts were chosen, and within each district, two kindergartens were randomly selected. Per kindergarten, 20 children (referred to as “index children”) and five staff members were recruited, if possible, from one care group. Household members of recruited children and staff were also invited to participate [3]. This cohort formed the basis for participation in our data collection in September 2021. Only participants with a negative antibody result in September 2020 were eligible for our antibody analysis in September 2021.

In September 2021, study teams visited each facility to collect samples and data. Kindergarten children, staff, and household members were interviewed on present symptoms of COVID-19. Finger-prick blood samples were collected on filter paper (Bio-Sample Card, Ahlstrom Munksjö, Bärenstein, Germany). Reverse transcriptase–polymerase chain reaction (Roche COBAS SARS-CoV-2 test) was used to determine SARS-CoV-2 infection. Additionally, participants were asked to fill in an electronic questionnaire using the Research Electronic Data Capture (REDCap) tool [22] on further parameters including sex, age, sociodemographic background, contact to positive cases, protection behavior, pre-existing medical conditions, medication, SARS-CoV-2 infection, and vaccination status. Parents answered the questionnaire for kindergarten children.

Antibodies were determined using Roche Elecsys^®^ Anti-SARS-CoV-2 test kits. For that, 3 × 3.2 mm discs were punched from dried blood spots and eluted for 1 h at 37 °C with 300 rpm shaking (MIUlab ES-60E, Hangzhou Miu Instruments, Hangzhou, China). As described elsewhere, antibodies were measured on cobas e801 modules (Roche, Mannheim, Germany) [23]. We considered only those participants for analysis who provided a blood sample of sufficient volume and had a confirmed antibody result [23].

We tested for both anti-S antibodies (detectable after infection and/or vaccination), and anti-N antibodies (detectable only after infection) [24,25], and categorized the antibody results in three groups: (i) negative, i.e., negative for anti-S and anti-N; (ii) vaccinated, i.e., only anti-S positive; and (iii) previously infected, i.e., positive for anti-N, or anti-S and anti-N. Since the vaccine was only approved for age groups 12 years and older, we assumed that children below 12 years of age showing only anti-S antibodies had previously been infected and already lost anti-N. This phenotype is plausible for mild infections where anti-N is dropping fast, or in case the anti-N reaction was delayed and had not yet taken place when the sample was taken [24,25]. For older participants with anti-S antibodies only (possibly vaccinated), we used the information from the questionnaires (SARS-CoV-2 infection and vaccination status) to identify individuals with a previous infection and lacking anti-N. These were also classified as previously infected. We categorized individuals as having undergone an undiagnosed infection if they were allocated to the serological group of “previously infected” but had not experienced a SARS-CoV-2 infection, according to their questionnaire. Participants were grouped as kindergarten children, staff, and household members and according to age for analysis. We calculated proportions for categorical variables and median (range) for continuous variables. To assess the associated factors with unrecognized SARS-CoV-2 infection and with preceding infections, we calculated crude odds ratios (OR) using cross-tabulation, and the corresponding 95% confidence intervals. Data were processed and analyzed using IBM SPSS Statistics 28.0.1.0 [26].

## 3. Results

### 3.1. Characteristics of Study Participants

Out of 580 BECOSS participants in September 2021, 546 had a seronegative antibody test result from the initial study visit one year earlier [3] and therefore fulfilled the eligibility criteria. In September 2021, 169 of those 546 individuals rejected antibody testing and thus did not provide a second blood sample. Of the 377 samples obtained, 71 had limited evaluability (mostly insufficient blood volume) and were excluded. For 29 participants, no information about infection history was provided. Hence, our final dataset included 277 participants with confirmed antibody tests and questionnaire information on infection history. Among those, 48 (17.3%) were kindergarten children, 37 (13.4%) were kindergarten staff, and 192 (69.3%) were household members (Table 1). All tested negative for SARS-CoV-2 using rt-PCR.

The kindergarten children had a median age of 5 years; more than half were male. Staff were largely female with a median age of 49 years. Household members consisted of pre-school children (siblings) (n = 16; median age, 3 years; range, 2–6), school children (n = 44; median age, 9 years; range, 7–18), and adults (n = 132; median age, 40 years; range, 24–79). Further characteristics are shown in Table 1. Leading signs and symptoms stated to have occurred within the preceding 24 h were headache, rhinitis, and cough, with the latter two occurring more common in daycare children (Table 1). No signs of severe acute disease were observed at presentation.

### 3.2. SARS-CoV-2 Antibodies

SARS-CoV-2 antibodies were detected in 65.0% (180/277) of the participants (Table 2). This combined 52.7% of individuals categorized as vaccinated and 12.3% as previously infected. Looking at the subgroups, 81.1% of staff and 60.4% of household members were considered as vaccinated. Categorization as previously infected applied to 16.7% of kindergarten children, 16.2% of staff, and 10.4% of household members.

Undiagnosed infections were found in 5.4% of all participants (Table 2). This figure was higher among kindergarten children (12.5%) as compared to household members (3.6%, crude OR 3.8; 95% confidence interval (CI), 1.2–11.8; Table 2). Grouping all children under 12 years of age (index kindergarten children and siblings from the household group), i.e., those ineligible for SARS-CoV-2 vaccination, the proportion of undiagnosed infections was almost five times higher (11.0%, 11/100) than in older participants (2.3%, 4/177; crude OR 5.3; 95% CI, 1.7–17.3). At the same time, among those categorized as previously infected, 44.1% (15/34) did not recall an episode of infection. This proportion was 75% (6/8) in kindergarten children, 33.3% (2/6) in staff, and 35% (7/20) in household members; or 64.7% (11/17) in children under 12 years and 23.5% (4/17) in participants aged 12 years or above (crude OR 6.0; 95% CI, 1.3–26.7).

Lastly, comparing individuals with serological and questionnaire-based evidence for preceding infection (n = 34) with those lacking such evidence (n = 243), the self-reported neglect of wearing a facemask was associated with preceding infection (crude OR, 3.7; 95%CI, 1.4–9.7; 57.9%, 11/19 vs. 27.3%, 45/165).

## 4. Discussion

In the present study assessing SARS-CoV-2 serostatus in a Berlin kindergarten environment in September 2021, two in three participants were SARS-CoV-2 seropositive, which was largely a result of vaccination among adults. Yet, one in six kindergarten children had non-vaccine-induced antibodies. The proportion of undiagnosed infections was highest in kindergarten children, and almost five times higher in children below 12 years of age than in persons aged 12 years or older. Facemask neglect was associated with previous SARS-CoV-2 infection.

Our identified SARS-CoV-2 seroprevalence of 16.7% in kindergarten children exceeds findings from other, mostly earlier studies in Germany. In a Bavarian screening for type 1 diabetes (Fr1da) during the second pandemic wave (September 2020–February 2021), a seroprevalence of 3.9% was observed in children aged 1–10 years [10,11]. In another Bavarian study in September 2021, seroprevalence was 11.8% in children 1–17 years [27]. Similar seroprevalences were found in children <18 years in Western Germany between June 2020 and February 2021 [12,28], or among >10,000 children admitted to hospitals across Germany in spring 2021 [29]. Regarding kindergarten staff, our identified seroprevalence of 16.2% exceeds results from France, where a multicenter study in 22 kindergartens showed a seroprevalence of 6.8% among kindergarten staff between March and May 2020 [30]. A study on transmission in daycare facilities in Germany showed that transmission was more likely to occur in households than in institutions [31]. In a meta-analysis from 2020, adults had a higher risk of infection than children in the same households [32]. This is supported by another meta-analysis from 2022, which shows that children do not dominate household transmission [33].

Seroprevalence studies provide important data on asymptomatic and unrecognized infections. This is corroborated by the fact that we found 16.7% seroprevalence in kindergarten children as compared to a 3.2% PCR-based cumulative infection frequency in children <5 years of age according to Robert Koch-Institute data in September 2021 in Germany [34], suggesting under-diagnosing in this age group. More undiagnosed infections in kindergarten children compared to older age groups could be caused by the fact that children develop fewer symptoms during SARS-CoV-2 infection [9,35,36,37,38,39,40,41]. The proportion of asymptomatic infections in children in previous research varied between 15% in China [9], 59% in Western Germany [12,28], and 47% to 68% in Bavaria [10,11]. Of note, none of our study participants tested positive for SARS-CoV-2 using rt-PCR. Fecal shedding of SARS-CoV-2, partially persistent, has been described in a substantial proportion of infected children [42] and associated with transmission [43]. However, the extent to which the detection of viral RNA in the stool of children is actually associated with transmission remains unclear [10,41]. Regardless, diarrhea is most common in SARS-CoV-2 infected pediatric outpatients at kindergarten age, which could facilitate transmission [44]. For future epidemic events, kindergarten staff need to take asymptomatic pediatric infections into account and employ counteracting measures like building fixed, small contact groups and check regularly for infections through pooled PCR testing even if no symptoms are present.

Preceding infection was associated with never wearing a facemask, supporting the known protective effect [12,45,46,47,48,49]. Other studies also reported low socioeconomic status or migration background to influence seropositivity among children [28,50]. In the present study, further associations were not observed, likely also due to its limited sample size. Kindergarten staff showed the highest vaccination rate (81.1%) in our study. A reason could be that kindergarten staff were a prioritized group for vaccination and could get vaccinated from April 2021 onwards, while most of the younger adults (e.g., parents) needed to wait until June 2021 [20].

One strength of our study is the random selection of kindergartens throughout Berlin. We generated empirical data and did not base our findings on model-based simulated estimates. Connected household members were also invited into the study. We used robust laboratory methods and performed non-symptomatic screening. However, our study also has limitations. First, voluntary participation entails a selection bias; possibly, participants who were vaccinated or who assumed an undiagnosed previous infection were more likely to participate in this serological survey. Secondly, we used dried blood spots for serology because of the less invasive finger-prick sampling [23,51,52,53]. The quality of such results can be hampered depending on blood volume [23,53,54], but to avoid that, we only included samples with sufficient volume and confirmed results. Another limitation is that antibodies can decrease over time to an undetectable level [25,38,55,56], and among vaccinated participants aged 12 years and older, previous infections could be masked by a comparatively faster decrease in anti-N antibodies than anti-S antibodies [24]. However, we triangulated serostatus with questionnaire information to reduce this effect. Nevertheless, the number of undiagnosed infections within our group could be an underestimation especially among vaccinated adolescents and adults. Finally, our findings must be seen in the light of the highly transmissible Omicron variant in late 2021, with recent German studies showing substantially increased seroprevalence in young children of up to 70% by May 2022 [27,57].

## 5. Conclusions

By September 2021, two-thirds of our tested cohort were SARS-CoV-2 seroreactive, largely as a result of vaccination in adults. One in six kindergarten children showed non-vaccine-induced seropositivity for SARS-CoV-2 and the highest proportion of undiagnosed infections in our kindergarten-based cohort. The high proportion of unrecognized infections in pre-school children needs to be considered when interpreting SARS-CoV-2 infections in the kindergarten context.

## Figures and Tables

**Table 1:** Characteristics of study participants, Berlin, September 2021.

Characteristics		All	Kindergarten Children	Kindergarten Staff	Household Members
N		277	48	37	192
Female (%, n/n)		53.8 (149/277)	41.7 (20/48)	91.9 (34/37)	49.5 (95/192)
Age (median, range)		36 (2–79)	5 (3–7)	49 (21–79)	38 (2–79)
Household education level (%, n/n) ^a^	High	60.1 (95/158)	64.7 (22/34)	44.4 (12/27)	62.9 (61/97)
	Middle	30.4 (48/158)	26.5 (9/34)	40.7 (11/27)	28.9 (28/97)
	Low	9.5 (15/158)	8.8 (3/34)	14.8 (4/27)	8.2 (8/97)
Household income level (%, n/n) ^b^	High	48.7 (75/154)	42.4 (14/33)	19.2 (5/26)	58.9 (56/95)
	Middle	46.1 (71/154)	51.5 (17/33)	69.2 (18/26)	37.9 (36/95)
	Low	5.2 (8/154)	6.1 (2/33)	11.5 (3/26)	3.2 (3/95)
Migration background (%, n/n) ^c^		11.3 (18/159)	17.6 (6/34)	7.4 (2/27)	10.2 (10/98)
Contact to confirmed or suspected SARS-CoV-2 infected case outside of kindergarten/ school/ work (%, n/n)		23.0 (43/187)	78.4 (29/37)	19.2 (5/26)	7.3 (9/124)
Never wearing facemask (%, n/n)		30.4 (56/184)	68.4 (26/38)	25.9 (7/27)	19.3 (23/119)
Pre-existing health condition (%, n/n)		18.0 (34/189)	5.3 (2/38)	37.0 (10/27)	17.7 (22/124)
Presence of signs and symptoms in last 24 h (%, n/n)	Fever	2.7 (5/187)	2.7 (1/37)	3.8 (1/26)	2.4 (3/124)
	Cough	20.1 (38/189)	34.2 (13/38)	18.5 (5/27)	16.1 (20/124)
	Sore throat	11.2 (21/187)	5.4 (2/37)	11.1 (3/27)	13.0 (16/123)
	Rhinitis	24.3 (46/189)	50.0 (19/38)	18.5 (5/27)	17.7 (22/124)
	Breathlessness	4.3 (8/187)	2.7 (1/37)	11.1 (3/27)	3.3 (4/123)
	Chest tightness	1.6 (3/184)	0 (0/36)	3.8 (1/26)	1.6 (2/122)
	Headache	25.7 (48/187)	8.1 (3/37)	33.3 (9/27)	29.3 (36/123)
	Fatigue	17.1 (32/187)	8.3 (3/36)	33.3 (9/27)	16.1 (20/124)
	Shivering	1.1 (2/187)	0 (0/36)	7.4 (2/27)	0 (0/124)
	Loss of smell and/or taste	3.2 (6/188)	0 (0/37)	0 (0/27)	4.8 (6/124)
	Limb pain	2.7 (5/187)	0 (0/37)	14.8 (4/27)	0.8 (1/123)
	Diarrhea	3.2 (6/186)	0 (0/37)	3.7 (1/27)	4.1 (5/122)

a. Person with the highest education in the household was counted, grouped as low (Intermediate school diploma), middle (university entry qualification), high (university degree). b. Total net household income, grouped as low (EUR 1000–2000), middle (EUR 2000–5000), high (EUR > 5000). c. Both grandparents of the kindergarten child not born in Germany.

**Table 2:** Anti-SARS-CoV-2 antibody prevalence in kindergarten children, kindergarten staff and household members, Berlin, September 2021.

		All	Kindergarten Children	Kindergarten Staff	Household Members
N		277	48	37	192
Anti-S positive (%, n/n)		64.6 (179/277)	16.7 (8/48)	97.3 (36/37)	70.3 (135/192)
Anti-N positive (%, n/n)		6.9 (19/277)	4.2 (2/48)	8.1 (3/37)	7.3 (14/192)
Categorized SARS-CoV-2 antibody result ^a^	Negative	35.0 (97/277)	83.3 (40/48)	2.7 (1/37)	29.2 (56/192)
	Vaccinated	52.7 (146/277)	0	81.1 (30/37)	60.4 (116/192)
	Previously infected	12.3 (34/277)	16.7 (8/48)	16.2 (6/37)	10.4 (20/192)
Proportion of undiagnosed SARS-CoV-2 infection (%, n/n) ^b^		5.4 (15/277)	12.5 (6/48)	5.4 (2/37)	3.6 (7/192)

a. Negative (SARS-CoV-2 anti-S and anti-N negative); vaccinated (SARS-CoV-2 only anti-S positive and age ≥12 years); previously infected (SARS-CoV-2 anti-S and anti-N positive, or with previous infection answered in the questionnaire, or children <12 years with only anti-S positive, and adults with only anti-S and never vaccinated). b. SARS-CoV-2 antibody results from participants who were categorized as previously infected but did not remember an infection. Five participants had anti-S and anti-N antibodies. Another 10 participants had anti-S only; of these, most were kindergarten children (6/10) and unvaccinated, in addition one adult household member also credibly affirmed never having been vaccinated. The 15 undiagnosed infections were distributed between the six different kindergartens (median 2.5 cases).

## Data Availability

Data are not stored publicly due to underage participants, but can be made available upon reasonable scientific request from the corresponding author.
